# Supplementary material for: Enolase Inhibitors as Early Lead Therapeutics against Trypanosoma brucei
Source: Pathogens. 2023 Oct 28;12(11):1290. doi: 10.3390/pathogens12111290 (PMC10675445; doi:10.3390/pathogens12111290)
Supplement: Supplementary file 1 [file pathogens-12-01290-s001.zip › pathogens-2627041-supplementary.pdf]

```
NfTy_054390         MSKANNSSTQAKKPQQKKVAKKVKKTLANPFSPKFKILSSEQQNKVLKLIQECPFHNIIY 60
Tb927.10.2890       ------------------------------------------------------------ 0
ENOG_Human,         ------------------------------------------------------------ 0


NfTy_054390         EVNRISEIISSPQSHQIELVIFPDANYLQINPIQIDFLINQCSYAKIPFINLEINVQSFT 120
Tb927.10.2890       ------------------------------------------------------------ 0
ENOG_Human,         ------------------------------------------------------------ 0


NfTy_054390         EVCKKHYSSLSLHPNNFLKAIAFESTHDDTSMDPQQSQYNILELLYQENVYGPSIPREDL 180
Tb927.10.2890       ------------------------------------------------------------ 0
ENOG_Human,         ------------------------------------------------------------ 0


NfTy_054390         FYVPPFISSSFQYEPAHVEDVSEYLQNHNLQKLVEDALNECYNANASDPVGFLGHFFLNR 240
Tb927.10.2890       ------------------------------------------------------------ 0
ENOG_Human,         ------------------------------------------------------------ 0


NfTy_054390         GKKGAVNRVDKLVGREILDSRGNPTVEVDVYANGQKRPVATASAPSGASTGSNEAHELRD 300
Tb927.10.2890       ------MTIQKVHGREVLDSRGNPTVEVEVTTE---RGVFRSAVPSGASTGVYEACELRD 51
ENOG_Human,         ------MSIEKIWAREILDSRGNPTVEVDLYTA---KGLFRAAVPSGASTGIYEALELRD 51
                            ::*: .**:***********:: :    : :  ::.*******  ** ****

NfTy_054390         GDKSRYLGKGVLKAVKNVNDVLGKAVEGKSLEN--LTELDQALIDADGDELKSNLGGNAI 358
Tb927.10.2890       GDKKRYVGKGCLQAVKNVNEVIGPALIGR--DELKQEELDTLMLRLDGTPNKGKLGANAI 109
ENOG_Human,         GDKQRYLGKGVLKAVDHINSTIAPALISSGLSVVEQEKLDNLMLELDGTENKSKFGANAI 111
                    ***.**:*** *:**.::*..:. *: .   .     :**  ::  **   *.::*.***

NfTy_054390         TACSFALATAGAAVRNEELFLYLARAFHGADKFENLKFRLPTPMVNILNGGKHAGGRLQI 418
Tb927.10.2890       LGCSMAISKAAAAAKGVPLYRYLASLA------GTKELRLPVPCFNVINGGKHAGNALPF 163
ENOG_Human,         LGVSLAVCKAGAAERELPLYRHIAQLA------GNSDLILPVPAFNVINGGSHAGNKLAM 165
                     . *:*:..*.** :   *: ::*          . .: **.* .*::***.***. * :

NfTy_054390         QEFMILPKENQPFREKVRCVAEVYQHLGKILAERAGPSAKNVGDEGGFAPNLETADEALN 478
Tb927.10.2890       QEFMIAPVKATSFSEALRMGSEVYHSLRGIIKKKYGQDAVNVGDEGGFAPPIKDINEPLP 223
ENOG_Human,         QEFMILPVGAESFRDAMRLGAEVYHTLKGVIKDKYGKDATNVGDEGGFAPNILENSEALE 225
                    ***** *     * : :*  :***: *  :: .: * .* ********** :   .* *

NfTy_054390         YIEEAIGKAGYKVGEDVFLAL------------------DAASKFLTSEEMVEYYVQLVN 520
Tb927.10.2890       ILMEAIEEAGHRGKFAICMDCAASETYDEKKQQYNLTFKSPEPTWVTAEQLRETYCKWAH 283
ENOG_Human,         LVKEAIDKAGYTEKIVIGMDVAASEFYRDGKYDLDFKSPTDPSRYITGDQLGALYQDFVR 285
                     : *** :**:     : :                         ::*.:::   * . ..

NfTy_054390         RHPAIISIEDGLEEKDYEGWKLLTERLGSKIMLVGDDLYTTNTRLIKQGIEEKWANALLL 580
Tb927.10.2890       D-YPIVSIEDPYDQDDFAGFAGITEALKGKTQIVGDDLTVTNTERIKMAIEKKACNSLLL 342
ENOG_Human,         D-YPVVSIEDPFDQDDWAAWSKFTANV--GIQIVGDDLTVTNPKRIERAVEEKACNCLLL 342
                        ::****  ::.*: .:  :*  :     :***** .** . *: .:*:* .*.***

NfTy_054390         KVNQIGTITEAMNAARMIFNVGQKVIVSHRSGETATTLISDLVVGIGATHIKTGATARGE 640
Tb927.10.2890       KINQIGTISEAIASSKLCMENGWSVMVSHRSGETEDTYIADLVVALGSGQIKTGAPCRGE 402
ENOG_Human,         KVNQIGSVTEAIQACKLAQENGWGVMVSHRSGETEDTFIADLVVGLCTGQIKTGAPCRSE 402
                    *:****:::**: :.::  : *  *:********  * *:****.: : :***** .*.*

NfTy_054390         RVSKYNRLLQIEEYLEQHGLLA----------     662
Tb927.10.2890       RTAKLNQLLRIEEELGAHAKFGFPGWS-----     429
ENOG_Human,         RLAKYNQLMRIEEELGDEARFAGHNFRNPSVL     434
                    * :* *:*::*** *  .. :.
```

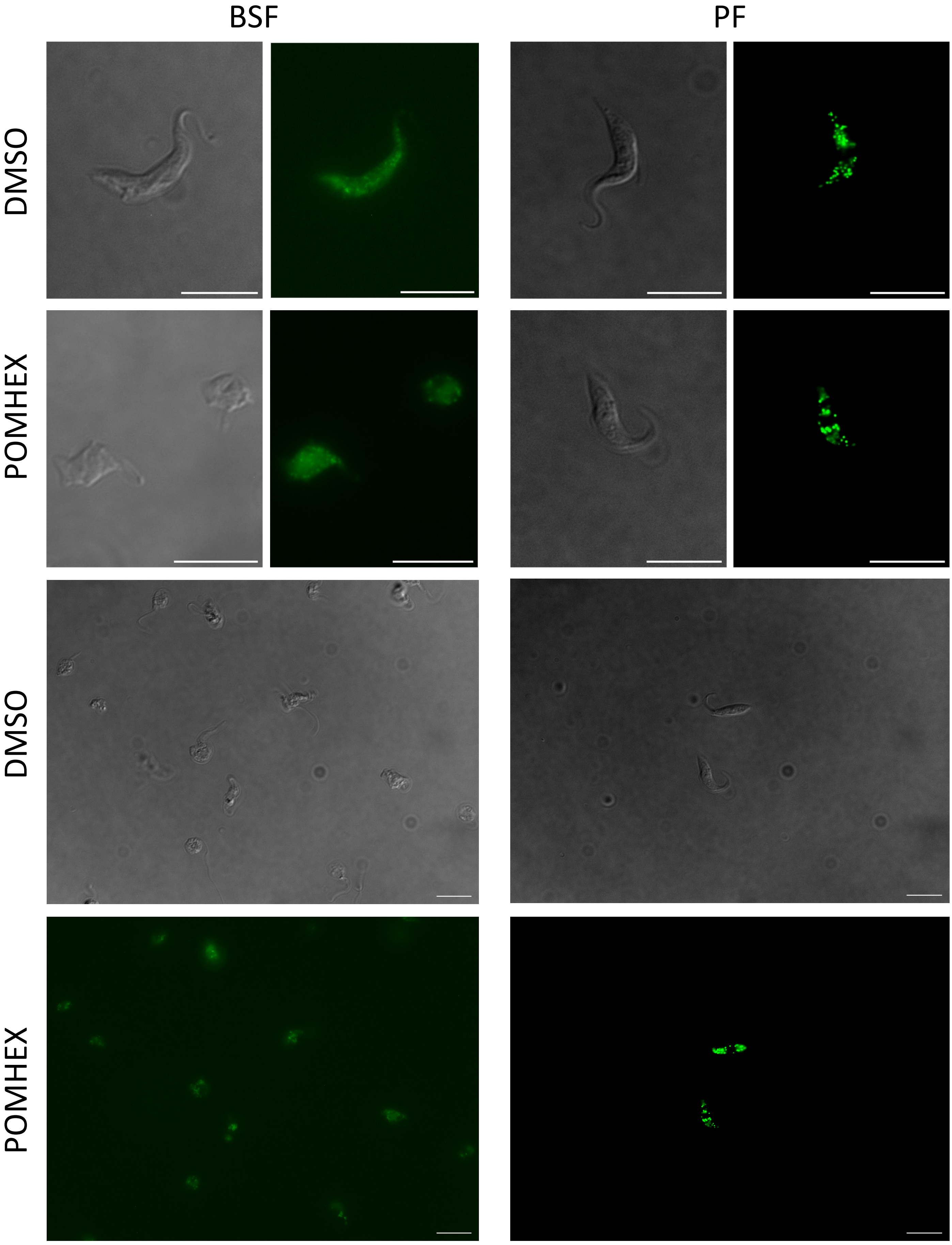
BSF
PF
DMSO
POMHEX
DMSO
POMHEX

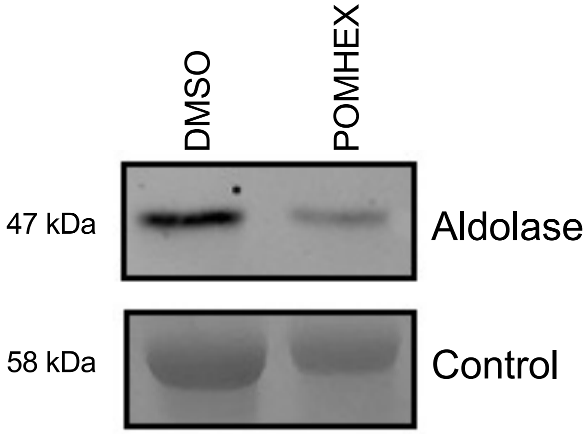
DMSO
POMHEX
47 kDa
Aldolase
58 kDa
Control

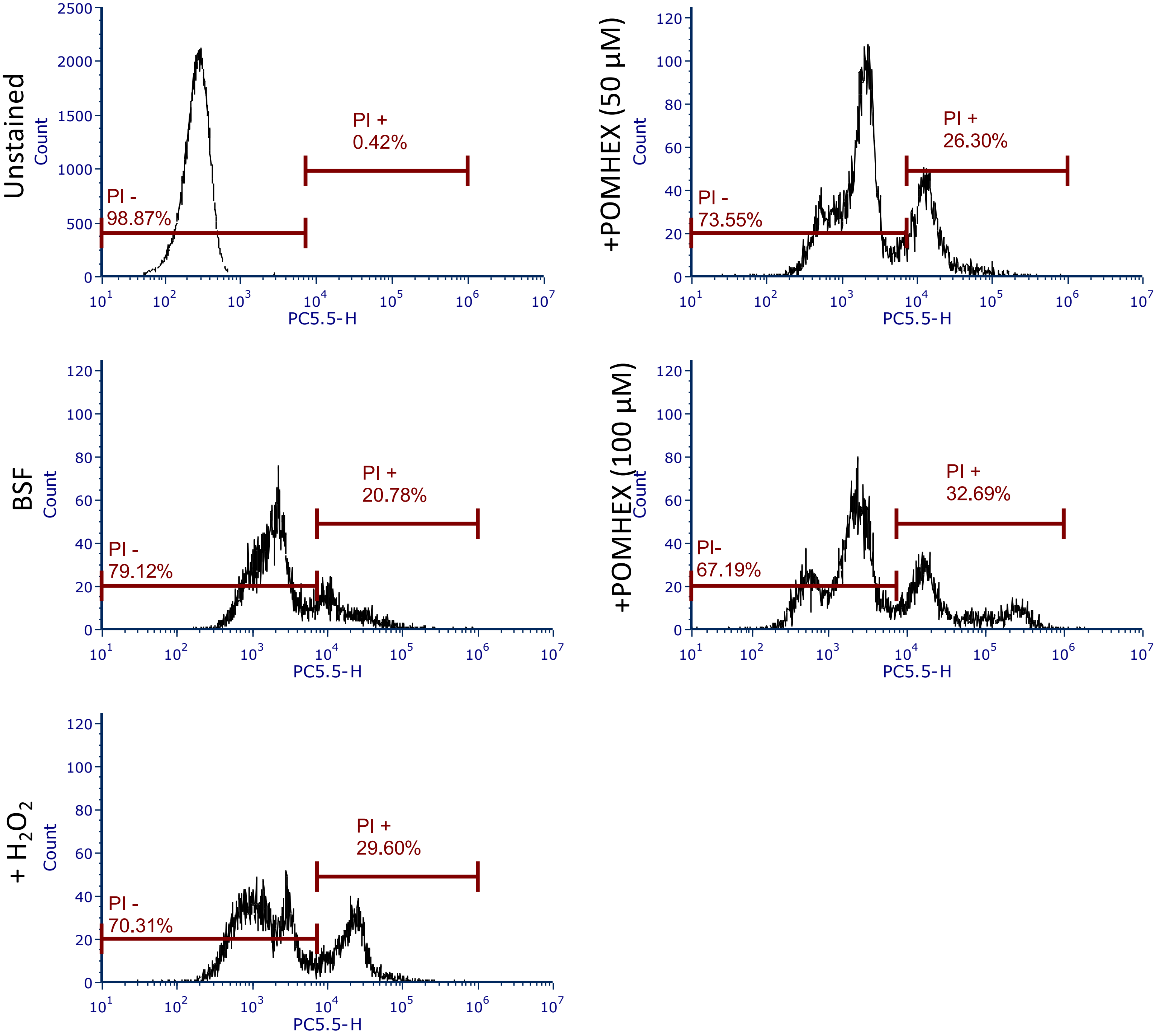
Unstained
Count
PI +
0.42%
PI -
98.87%
PC5.5-H
+POMHEX (50 μM)
PI +
26.30%
PI -
73.55%
BSF
PI +
20.78%
PI -
79.12%
+POMHEX (100 μM)
PI +
32.69%
PI-
67.19%
+ H2O2
PI +
29.60%
PI -
70.31%
